# Emerging Therapies in Pheochromocytoma and Paraganglioma: Immune Checkpoint Inhibitors in the Starting Blocks

**DOI:** 10.3390/jcm10010088

**Published:** 2020-12-29

**Authors:** Giuseppe Fanciulli, Sergio Di Molfetta, Andrea Dotto, Tullio Florio, Tiziana Feola, Manila Rubino, Federica de Cicco, Annamaria Colao, Antongiulio Faggiano, NIKE Group

**Affiliations:** 1 NET Unit, Department of Medical, Surgical and Experimental Sciences, University of Sassari—Endocrine Unit, AOU Sassari, 07100 Sassari, Italy; 2 Department of Emergency and Organ Transplantation, Section of Internal Medicine, Endocrinology, Andrology and Metabolic Diseases, University of Bari Aldo Moro, 70124 Bari, Italy; s.dimolfetta@libero.it; 3 Endocrinology Unit, IRCCS Ospedale Policlinico San Martino, 16132 Genova, Italy; andreadotto91@gmail.com; 4 Department of Internal Medicine, University of Genova, 16132 Genova, Italy; tullio.florio@unige.it; 5 IRCCS Ospedale Policlinico San Martino, 16132 Genova, Italy; 6 Department of Clinical and Molecular Medicine, Sapienza University of Rome, 00161 Rome, Italy; tiziana.feola@uniroma1.it (T.F.); antongiulio.faggiano@uniroma1.it (A.F.); 7 Neuroendocrinology, Neuromed Institute, IRCCS, 86077 Pozzilli (IS), Italy; 8 Division of Gastrointestinal Medical Oncology and Neuroendocrine Tumors, European Institute of Oncology, IEO, IRCCS, 20141 Milan, Italy; manila.rubino@ieo.it; 9 Department of Clinical Medicine and Surgery, Endocrinology Unit, University Federico II, 80131 Naples, Italy; deciccofederica25@gmail.com (F.d.C.); colao@unina.it (A.C.)

**Keywords:** pheochromocytoma, paraganglioma, immune checkpoint inhibitors, avelumab, atezolizumab, ipilimumab, nivolumab, pembrolizumab, cemiplimab, durvalumab

## Abstract

Pheochromocytoma and paraganglioma are neuroendocrine neoplasms, originating in the adrenal medulla and in parasympathetic and sympathetic autonomic nervous system ganglia, respectively. They usually present as localized tumours curable with surgery. However, these tumours may exhibit heterogeneous clinical course, ranging from no/minimal progression to aggressive (progressive/metastatic) behavior. For this setting of patients, current therapies are unsatisfactory. Immune checkpoint inhibitors have shown outstanding results for several types of solid cancers. We therefore aimed to summarize and discuss available data on efficacy and safety of current FDA-approved immune checkpoint inhibitors in patients with pheochromocytoma and paraganglioma. After an extensive search, we found 15 useful data sources (four full-published articles, four supplements of scientific journals, seven ongoing registered clinical trials). The data we detected, even with the limit of the small number of patients treated, make a great expectation on the therapeutic use of immune checkpoint inhibitors. Besides, the newly detected predictors of response will (hopefully) be of great helps in selecting the subset of patients that might benefit the most from this class of drugs. Finally, new trials are in the starting blocks, and they are expected to shed in the next future new light on a therapy, which is considered a milestone in oncology.

## 1. Introduction

Pheochromocytoma (PHEO) and paraganglioma (PGL) are neuroendocrine neoplasms (NENs), originating in the adrenal medulla and in parasympathetic and sympathetic autonomic nervous system ganglia, respectively.

Most patients with PHEO and PGL present with localized tumours that are curable with surgery. However, these tumours exhibit heterogeneous clinical course, ranging from no/minimal progression to aggressive (progressive/metastatic) behavior, [1], with high morbidity and mortality rates [2].

For this subgroup of patients, several therapeutic strategies for control of tumour burden have been evaluated, and/or clinical trials are ongoing.

Therapeutic options include radionuclide therapy, such as 131-iodinemetaiodobenzylguanidine (MIBG) [3]; iobenguane, a high-specific activity preparation of 131-ioidine-MIBG, that is the first and only therapy approved by FDA for patients with locally advanced or metastatic PHEO/PGL [4]; peptide receptor radionuclide therapy (i.e., somatostatin analogs labeled with 177-lutetium or 90-yttrium) [5], and chemotherapy (i.e., cyclophosphamide, vincristine, and dacarbazine) [6]. Some favorable results have also been obtained with somatostatin analogues [7], temozolomide [8], sunitinib [9], and everolimus [10].

In recent years, immunotherapy has provided promise for revolutionizing cancer treatment through the implementation of new approaches that enhance the body’s natural antitumour defenses.

Although the immune system actively participates in the control of tumour development and growth through adaptive and immune responses that favor the elimination of neoplastic cells, with time tumour cells acquire the capacity to escape immune surveillance. This process, named cancer immunoediting [11], involves the selection of subpopulations of tumour cells able to directly inhibit immune cell activity allowing tumour mass to grow. Among the different mechanisms responsible of the tumour-mediated immunosuppression, the modulation of immune checkpoint protein activity is nowadays considered central to tumour immune evasion, and, consequently a major pharmacological target [12].

Immune system ability to recognize and eliminate potential noxious situations, including tumour cells, requires the activation of T cells. In particular, during tumour development, immune response is activated by the binding of T-cell receptor (TCR), expressed by T cells, and tumour associated antigens (TAA) within MHC molecules, via the activity of antigen-presenting cells (APCs), such as dendritic cells. This initial T lymphocyte activation mainly occurs within lymph nodes, during the first phase of the T cell-mediated immune response (priming). However, T cell activation after antigen binding requires the induction of a second signaling pathway mediated by the interaction of B7-1 and B7-2 ligands (also known as CD80 and CD86), expressed by APCs, and the CD28 co-receptor, expressed on T cells. This interaction induces T cell proliferation but, if it is blocked during TCR activation, T cells undergo to inactivation and, ultimately, apoptosis. This represents a first immune checkpoint since, to avoid immune cells overstimulation, CD28 binding to B7-1 or B7-2 can be antagonized by the interaction with cytotoxic T lymphocyte antigen-4 (CTLA-4) expressed by lymphocytes [13]. The absence of the CD28 signaling caused by CTLA-4 binding leads to T cell anergy. In cancer, CTLA-4 expression is induced in T cells after TCR activation and its signaling starts when APCs migrate from cancer to draining lymph nodes, here presenting the tumour-associated antigen to T lymphocytes, replacing CD28 by the interaction with B7 ligands [14]. Importantly, besides activated T cells, CLTA-4 is also expressed on regulatory T-cells contributing to the inhibition of the antitumour immune response. On this premises, CTLA-4 blockade was proposed as novel anticancer immunotherapy [15].

A second immune checkpoint, involving programmed cell death-1 (PD-1) inhibitory signals, was subsequently discovered in peripheral tissues [16], and relevant to the antitumour response suppression, in cells composing tumour microenvironment. PD-1 is mainly expressed on effector T lymphocytes, but can also be detected in NK cells. Its binding by two ligands (programmed death-ligand-1, PD-L1; programmed death-ligand-2, PD-L2) inhibits activation signals in T cells. Physiologically this response is considered a way to modulate immune response preventing autoimmunity. PD-L1 (also known as B7-H1) is expressed by diverse cell types, including neurons and astrocytes, endothelial/epithelial cells, and pancreatic islet cells, while PD-L2 is more confined to APCs. The immunosuppressive role of PD-1 signaling was also demonstrated by its overexpression in exhausted T lymphocytes, a state of functional unresponsiveness of T cells, that occur after sustained activation, for example in cancer patients [17].

As far as cancer cells, overexpression of PD-L1 is commonly observed both in primary tumours and in metastatic tissues, and its interaction with PD-1 leads to memory T cells inhibition [18]. Consequently, it was proposed that the blockade of PD-1/PD-L1 interaction could be a means to induce the reacquisition of antitumour activity by T cells. Moreover, PD-L1 was also reported to interact with B7-1, further potentiating its immunosuppressive activity [19].

**Figure 1:**
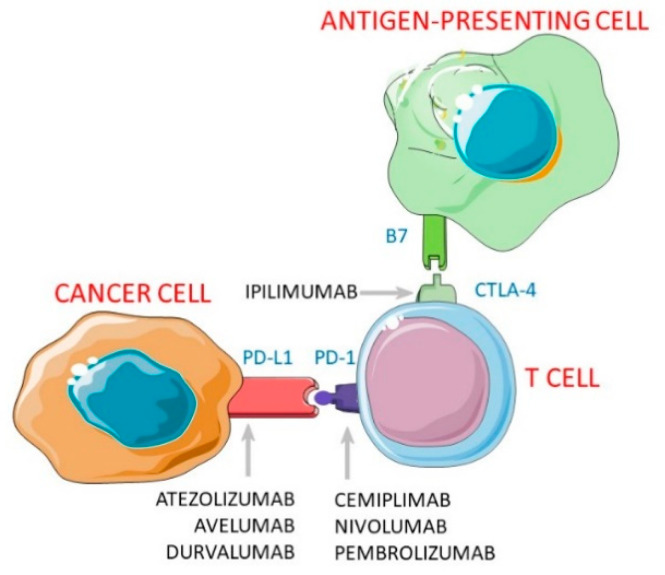
Molecular targets of Immune Checkpoint Inhibitors. Legend: PD-1: Programmed Cell Death-1; PD-L1: Programmed Death-Ligand-1; CTLA-4: Cytotoxic T Lymphocyte Antigen-4.

To date, several antibodies have been developed to interfere with both immune checkpoints, and approved for the treatment of several solid tumours. In particular, ipilimumab is an anti-CTLA-4 antibody, nivolumab, pembrolizumab and cemiplimab are anti PD-1 antibodies, and avelumab, atezolizumab, and durvalumab are anti PD-L1 antibodies (Figure 1).

In 2017, Pinato et al. have for the first time reported the programmed cell death ligands expression in PHEOs/PGLs with a distinctive prognostic, clinic-pathologic and immuno-biologic role, thus suggesting a potential therapeutic role for PD-1/PD-L1 targeted checkpoint inhibitors in these tumours [20].

### Aim of the Study

The aim of this review is to summarize and discuss available data on efficacy and safety of current FDA-approved immune checkpoint inhibitors (ICIs) in patients with PHEO/PGL.

List of drugs:

(1) Avelumab;
(2) Atezolizumab;
(3) Ipilimumab;
(4) Nivolumab;
(5) Pembrolizumab;
(6) Cemiplimab;
(7) Durvalumab.

Pharmacokinetic properties of ICIs are summarized in Table 1.

## 2. Materials and Methods

### 2.1. Published Articles

We performed an extensive search on PubMed, Embase, Cochrane Library, Web of Science, Scopus, by using the terms: avelumab, atezolizumab, ipilimumab, nivolumab, pembrolizumab, cemiplimab, durvalumab, paraganglioma, pheochromocytoma, neuroendocrine neoplasm, solid tumours. We additionally searched on Google Scholar in order to detect possible preliminary/partial data (published as supplement of scientific Journals). The search was last updated 23 November 2020.

### 2.2. Registered Clinical Trials (RCTs)

By using the same keywords used for the published articles, we performed an in-depth search on the following Registries: Clinical Trials Gov, EudraCT, and Chinese Clinical Trial Registry. The search was last updated 23 November 2020.

## 3. Results

### 3.1. Published Articles

We found 3254 published articles. Among them, we detected four pertinent clinical studies. We additionally found four short articles published as supplements of scientific journals.

### 3.2. RCTs

We found 952 RCTs, seven of which matched the aim of the study (treatment of PHEO/PGL with one or more of the considered drugs). We identified one study with avelumab (Phase I, recruiting), one with atezolizumab (Phase II, recruiting), two with ipilimumab and nivolumab in combination (Phase II and Phase II, recruiting), one with nivolumab (phase I/II, recruiting), two with pembrolizumab (Phase II, recruiting and Phase I/II, recruiting). Details of the RCTs are summarized in Table 2.

#### 3.2.1. Avelumab

Avelumab is a fully human, PD-L1 blocking, lambda immunoglobulin (Ig) G1 monoclonal antibody with potential antibody-dependent cell-mediated cytotoxicity [21].

The drug has been approved by FDA as a single agent for the treatment of metastatic Merkel cell carcinoma and locally advanced/metastatic urothelial carcinoma, and is also indicated as a first-line combination therapy with tyrosine kinase inhibitor axitinib in patients with advanced renal cell carcinoma [22].

Fatigue, musculoskeletal pain, diarrhea, nausea, infusion-related reaction, rash, decreased appetite, peripheral edema, and urinary tract infection were common adverse reaction (ARs) (i.e., reported in ≥20% of patients) when avelumab was administered as a single agent in registration trials [22]. To date, there is no published data on the efficacy and safety of avelumab in patients with PHEO/PGL.

The search on RCTs revealed 1 eligible study. *NCT02923466* (Phase 1 Trial of Vesicular Stomatitis Virus Genetically Engineered to Express NIS and Human Interferon Beta (VSV-IFNβ-NIS) Monotherapy and in Combination With Avelumab, in Patients With Refractory Solid Tumors) is a Phase 1 study aimed to evaluate in oncologic patients affected either by metastatic colorectal cancer, NEN or PHEO (estimated enrollment: 114 participants), the maximum tolerated dose (MTD) of VSV-IFNβ-NIS (VV1) in monotherapy and in combination therapy with avelumab (only one defined outcome). According to the protocol, participants aged 18 years and older with refractory solid tumours in arm III receive avelumab intravenously every 2 weeks starting on day 1, together with VV1 either intratumourally, intravenously or both. The study started in April 2017, with the estimated study completion date being February 2021. The present study status is “Recruiting”.

VV1 is an oncolytic virus engineered to selectively replicate in and kill human cancer cells, in patients with refractory advanced/metastatic solid tumours, including patients with adrenal medullary tumours. Therapy with VV1 consists of two main mechanisms. First, it selectively infects, replicates in and kills cancer cells; second, the lysis of these cells cause the release of cancer immunogenic antigens, with consequent immune system’s response [23]. Anti-PD-1 and anti-PD-L1 monoclonal antibodies, such as avelumab, are supposed to boost up and enhance said immune response.

Preliminary data of *NCT02923466* have been recently published [24]. Among the first 18 patients with solid tumours under study, 2 (11% of the population) had PHEO. The Authors identified the recommended VV1 dose, and compared three different VV1 infusion durations (monotherapy). No difference in safety between the three infusion durations (30, 60 and 180 min) was reported, with the better anti-tumour effect (efficacy) observed with the 30-min infusion.

#### 3.2.2. Atezolizumab

Atezolizumab is a humanized IgG1 kappa monoclonal antibody that selectively binds to PD-L1 and prevents its interaction with both PD-1 and B7-1 [25]. Atezolizumab has been approved by FDA as a single agent for the treatment of adult patients with locally advanced/metastatic urothelial carcinoma and metastatic non-small cell lung cancer (NSCLS), and is indicated in combination with other agents as a first-line treatment in selected patients with metastatic non-squamous NSCLC and triple-negative breast cancer [26].

Based on FDA prescribing information, fatigue, nausea, constipation, cough, dyspnea, and decreased appetite are common (≥20%) ARs when atezolizumab is administered as a single agent [26].

To date, there is no published data on the efficacy and safety of atezolizumab in patients with PHEO/PGL. 

The search on RCTs revealed 1 single study, named “Exploratory Basket Trial of Cabozantinib Plus Atezolizumab in Advanced and Progressive Neoplasms of the Endocrine System. CABATEN Study” (*NCT04400474*). This is a multi-cohort Phase 2 study aimed to evaluate, in the above mentioned population, including PHEO and PGL (estimated enrollment: 144 participants), the objective response rate (ORR, primary outcome), the adverse events (AEs), the duration of response (DoR), the progression-free survival (PFS), and the overall survival (OS) as secondary outcomes. According to the protocol, participants aged 18 years and older with advanced and progressive endocrine neoplasms receive atezolizumab 1200 mg intravenously every three weeks (cycle), together with cabozantinib 40 mg once daily. The study started in October 2020, with the estimated study completion date being March 2024. The present study status is “Recruiting”.

#### 3.2.3. Ipilimumab 

Ipilimumab is a fully human IgG1 kappa monoclonal antibody that binds with high affinity and inhibits CTLA-4 [27]. As a single agent, ipilimumab is approved for the treatment of patients with unresectable/metastatic melanoma, or patients with cutaneous melanoma and pathologic involvement of regional lymph nodes who have undergone a complete resection, including total lymphadenectomy. It is also indicated in combination with nivolumab for the treatment of intermediate/poor-risk patients with previously untreated advanced renal cell carcinoma [28].

Common AR (i.e., reported in >5% of patients) with ipilimumab as a single agent are fatigue, diarrhea, pruritus, rash, and colitis. Additional AR at high doses include nausea, vomiting, headache, weight loss, pyrexia, decreased appetite, and insomnia [28].

To date, there is no published data on the efficacy and safety of ipilimumab in patients with PHEO/PGL.

The approval of ipilimumab in combination with nivolumab for the majority of indications is linked with the proven enhanced antitumour response of the association of drugs compared with monotherapy alone in these tumours [29,30,31]. Ipilimumab and nivolumab, in fact, perform their immune checkpoint-inhibiting activity through different targets: the first one, as already said, inhibiting CTLA-4 activity, the latter blocking the interaction between PD-1 and its ligands, resulting in a complementary effect. Consistent with such evidence, currently ongoing trials evaluating ipilimumab activity for PHEO/PGL, are all designed in combination with nivolumab.

The search on RCTs revealed two eligible studies. *NCT02834013* (DART: Dual Anti-CTLA-4 and Anti-PD-1 Blockade in Rare Tumors) is a Phase 2 study of the combination of ipilimumab + nivolumab (arm I) vs. nivolumab alone (arm II) aimed to evaluate in patients affected by rare solid tumours, including PHEO and PGL (current estimated enrollment: 818 participants, original estimated enrollment: 334 participants), the ORR (primary outcome), the AEs, the BR, the clinical benefit rate (CBR), the OS, and the PFS (secondary outcomes). According to the protocol, participants aged 18 years and older with histologically and/or biochemically confirmed rare cancer, either in progression after at least one line of standard systemic therapy or lacking any other standard treatment proven to prolong survival, in arm 1 receive intravenous nivolumab over 30 min on days 1, 15, and 29 and intravenous ipilimumab over 60 min on day 1 every 42 days for up to 17 cycles, whereas in arm 2 receive only intravenous nivolumab over 30 min on days 1, 15, and 29. The study started in January 2017. The estimated study completion date is not given. The present study status is “Recruiting”.

*NCT03333616* (A Phase II Study of Nivolumab Combined With Ipilimumab for Patients With Advanced Rare Genitourinary Tumors) is a Phase 2 study aimed to evaluate in the above mentioned population, including PHEO/PGL (estimated enrollment: 57 participants), the ORR (primary outcome), the DoR, the PFS, the OS, and the AEs (secondary outcomes). According to the protocol, participants aged 18 years and older with advanced or metastatic urogenital cancer, receive intravenous nivolumab and ipilimumab every 3 weeks for a total maximum of four doses. After combination therapy, nivolumab is administered as monotherapy every 4 weeks. Doses are determined per protocol. The study started in December 2017, with the estimated study completion date being May 2025. The present study status is “Recruiting”.

Very preliminary data of *NCT03333616* have been recently published [32,33]: out of the 56 patients enrolled (median follow up 9.9 months, range <1–21), 18 (32.1%) were affected by adrenal tumours, but only two had PGL (3.6%). Of these two patients, one had stable disease (SD) and one had progressive disease (PD).

#### 3.2.4. Nivolumab

Nivolumab is a fully human monoclonal antibody of IgG4 kappa isotype, which binds to the PD-1 receptor and blocks its interaction with PD-L1 and PD-L2 [34]. Nivolumab is indicated for the treatment of a number of types of cancer including melanoma (both as a monotherapy and in combination with ipilimumab), renal cell carcinoma (in combination with ipilimumab), relapsed or refractory NSCLC, classical Hodgkin lymphoma, squamous cell cancer of the head and neck, colon cancer, liver cancer, and urothelial carcinoma as monotherapy [34].

When used as a single agent in registration trials, common (>20%) ARs were fatigue, rash, musculoskeletal pain, pruritus, diarrhea, nausea, asthenia, cough, dyspnea, constipation, decreased appetite, back pain, arthralgia, upper respiratory tract infection, pyrexia, headache, abdominal pain, and vomiting [35].

To date, there is no published data on the efficacy and safety of nivolumab in patients with PHEO/PGL.

As reported in the previous paragraph, nivolumab has been the object of study in two different RCTs in combination with ipilimumab, in order to maximize the immune checkpoint-blocking effect of the two molecules.

Regarding RCTs on nivolumab alone, the search on RCTs revealed 1 eligible study.

*NCT04187404* (A Phase 1/2 Trial of a Novel Therapeutic Vaccine (EO2401) in Combination With Immune CheckPoint Blockade, for Treatment of Patients With Locally Advanced or Metastatic Adrenocortical Carcinoma, or Malignant Pheochromocytoma/Paraganglioma) is a Phase 1/Phase 2 study aimed to evaluate in the above mentioned population (estimated enrollment: 72 participants), the AEs assessment (primary outcome), the OS, and the immunogenicity (secondary outcomes) of the four components that compose EO2401 (assessed by Interferon-γ ELISpot) after therapy with an innovative cancer peptide therapeutic vaccine in combination with nivolumab. According to the protocol, participants aged 18 years and older with histologically confirmed unresectable/metastatic adrenocortical carcinoma (ACC), or unresectable malignant PHEO/PGL, are grouped in five different cohorts and receive EO2401 in combination with nivolumab at standard dose. The recommended dose of EO2401 is to be found in Cohort 1 and will be administered to the other cohorts of patients, respectively composed by previously treated ACCs, previously untreated ACCs, previously treated PHEO/PGL, and previously untreated PHEO/PGL. The study started in July 2020, with the estimated study completion date being March 2024. The present study status is “Recruiting”.

The rationale of this study is based on the significant homologies existing between the TAAs and the onco-mimics, immunogenic, microbiome-derived peptides that will be administered in combination with nivolumab. As the presented antigens display high molecular homology with selected TAAs on adrenal cancer cells, a T-cell-mediated immune response is supposed to be mounted against the TAAs. Nivolumab here plays a fundamental role, permitting the adequate mounting of the T-cell response by allowing immune cell development and survival, therefore enhancing the immune cell-mediated anticancer effect.

This study is currently recruiting, but no result is available yet. Nonetheless, its conception certainly draws further attention to the possible role of immune-checkpoint blocking molecules also for the potential association with cancer peptide therapeutic vaccines.

#### 3.2.5. Pembrolizumab 

Pembrolizumab is a humanized monoclonal antibody of IgG4-kappa isotype that binds with high affinity to the PD-1 cell surface receptor [36]. On the basis of durable objective responses and a favorable safety profile, pembrolizumab has been approved for the treatment of melanoma, both small and NSCLS, head and neck squamous cell carcinoma, classic Hodgkin lymphoma, primary mediastinal large B-cell lymphoma, and diverse malignancies originating from either the gastrointestinal and urogenital tract [37].

In pivotal trials, common (≥20%) ARs for patients taking pembrolizumab as a single agent were fatigue, musculoskeletal pain, decreased appetite, pruritus, diarrhea, nausea, rash, pyrexia, cough, dyspnea, constipation, pain, and abdominal pain [37].

Evidence supporting the use of pembrolizumab in patients with PHEO/PGL is still limited. Naing et al. first reported data from a single-center, phase 2 trial (*NCT02721732,* Phase II Study for the Evaluation of Efficacy of Pembrolizumab (MK-3475) in Patients With Rare Tumors) with the aim to assess the efficacy and safety profile of pembrolizumab in adults with advanced rare cancers [38]. Final results from the cohort of patients with PHEO/PGL have been recently published [39]. Indeed, 11 patients with histopathological diagnosis of non-resectable metastatic PHEO/PGL, radiographic confirmation of metastases, and disease progression for 6 months prior to study start were included in the trial and received at least one dose of the investigational drug. Pembrolizumab was administered as a single 200 mg intravenous dose on the first day of every 21-day treatment cycle until patients either exhibited disease progression, developed unacceptable side effects, withdrew from the trial, or completed 24 months of treatment with the investigational drug. 

Non-progression rate (NPR) and incidence of AEs were the primary endpoints of the trial, the first one defined as the number of patients who were alive and progression free at 27 weeks, based on immune-related RECIST (irRECIST) criteria [40]. Predefined secondary endpoints included: (i) ORR; (ii) CBR for ≥4 months; (iii) PFS; (iv) OS; and (v) safety.

A single patient discontinued treatment early because of liver toxicity and was excluded from the primary (but not from secondary) endpoint analysis population.

Accordingly, NPR was 40% (four out of 10 evaluable patients), ORR was 9% (one out of 11 patients, with partial response (PR) as their best response, BR), and CBR was 73% (one out of 11 patients, 9%, with PR; seven out of 11 patients, 64%, with SD).

The median follow-up time was 17.9 months (IQR: 9.3–20.2 months). Seven patients exhibited radiographic evidence of PD, and one other patient had clinical progression, with the median PFS being 5.7 months (95% CI: 4.37—not reached). Six patients died because of disease progression across the trial, and the median OS was 19 months (95% CI: 9.9—not reached).

Safety data were consistent with the established profile of the drug. Four patients had grade 3 AEs, and no patient reported an adrenergic crisis during or immediately after the infusion [39].

In the same study, in search of possible markers of response to treatment with ICIs, PD-L1 membrane expression and presence of tumour infiltrating lymphocytes (TILs) were assessed in tumour samples of 9 out of 11 patients enrolled in the trial.

PD-L1 membrane expression was evaluated by immunohistochemistry, and an H-score ranging from 0 to 300 was assigned. TILs within tumour nests were microscopically evaluated and scored on a scale from 0 (absence of TILs) to 3 (intense intratumoural lymphocytic infiltration).

Overall, there was no clear association between PD-L1 membrane expression and TILs in primary tumour samples and clinical or radiological response [39].

In another study, Tapia et al. assessed tumour cell content (TC) both at baseline and between days 15 and 21 of the first cycle of treatment in 57 patients with advanced rare tumours from the abovementioned phase II trial [41]. TC was estimated on hematoxylin and eosin-stained slides and tumours were dichotomized into low and high TC according to a cut-off of 10%. Out of five patients with PHEO/PGL included in this analysis, four had high TC at baseline, and three had high TC on-treatment. In the total cohort, objective response was significantly associated with decrease in TC from baseline to on-treatment. However, data on the PHEO/PGL subgroup are not available.

Preliminary data on the role of radiomics in predicting RECIST-based tumour response to pembrolizumab [42] are available for 58 patients with either squamous cell carcinoma of the skin, ACC, carcinoma of unknown primary, and PGL (five patients [43]). By using selected radiomic features, the authors obtained a classification model able to distinguish between the 21 responders and the 37 non-responders of the study, with 100% sensitivity and 95% specificity. 

In late 2019, an interim analysis of a Phase I-II trial (*NCT02332668*) named KEYNOTE-051 has been published showing that Pembrolizumab has low antitumour activity in the majority of pediatric tumour types, and PD-L1 expression *per se* is not sufficient as a biomarker for response to treatment in pediatric patients [44]. The trial aims at investigating MTD/maximum administered dose, and dose confirmation in Phase I, and evaluating the safety and efficacy (for pediatric neoplasms) in Phase II (estimated enrollment: 310 patients). The actual study start date was in March 2015 and final results of KEYNOTE 051 are expected by September, 2022. To date, a single patient with PGL, located in the central nervous system, has been enrolled in the trial and therefore subjected to administration of pembrolizumab.

The primary hypothesis of this study was that administration of Pembrolizumab in PD-L1 positive tumours would result in an ORR greater than 10% for at least one type of neoplasm. According to the initial results thus far posted (154 patients), the only pediatric tumours where pembrolizumab showed significant activity was Hodgkin Lymphoma (ORR 60.0%). This trial was designed to include a wide variety of tumours histotypes, so that the numerousness of the groups of the same type of tumour is often low or very low. Conclusions regarding the antitumour efficacy of pembrolizumab are to be drawn considering the totality of patients, therefore no possible finding is obtainable for each subgroup, let alone the PGL one.

#### 3.2.6. Cemiplimab

Cemiplimab is a fully human monoclonal antibody of IgG4 kappa isotype that binds to the PD-1 receptor and blocks its interaction with both PD-L1 and PD-L2. Cemiplimab has been approved by FDA as a single agent for the treatment of adult patients with metastatic or locally advanced cutaneous squamous cell carcinoma who are not candidates for performing curative-intent surgery or radiation therapy. Based on drug labelling information, common (≥20%) ARs for cemiplimab are fatigue, rash and diarrhea [45]. To our knowledge, neither clinical studies have been published, nor have RCTs yet been registered for cemiplimab in this subset of patients.

#### 3.2.7. Durvalumab

Durvalumab is an engineered fully human, IgG1-kappa, monoclonal antibody directed against PD-L1, thus preventing its interaction with both PD-1 and CD80 [46]. The drug has been approved by FDA for the treatment of patients with locally advanced or metastatic urothelial carcinoma who had disease progression despite first-line platinum-based chemotherapy, or patients with unresectable, stage III NSCLC whose disease has not progressed following platinum-based chemotherapy and radiation therapy.

Common (>15%) ARs in registration trials were fatigue, musculoskeletal pain, constipation, decreased appetite, nausea, peripheral edema, urinary tract infection, cough, pneumonitis/radiation pneumonitis, upper respiratory tract infections, dyspnea, rash and alopecia [47]. As for cemiplimab, we did not find any published or ongoing study finalized at evaluating the efficacy on PHEO/PGL.

## 4. Discussion

Our review shows a substantial and increasing interest in the field of treatment of aggressive (progressive/metastatic) PHEO/PGL with ICIs. Even if the full results (in term of clinical/radiological outcomes) arise from the studies with pembrolizumab, very preliminary data are coming for RCTs involving avelumab [24], and ipilimumab + nivolumab [32,33]. 

As for pembrolizumab, the study performed by Jimenez and et al. [39] shows a 40% NPR, with a 73% of CBR, (being the 64% of SD the most frequent response), and a 19 months of OS, coupled with an acceptable safety profile. Despite the low number of subjects treated (11 patients), and the relatively short follow up (median, 17.9 months), the results appear promising, especially in view of the clinically severe status of the patients enrolled in this study, such as unresectable primary metastatic tumour, disease progression prior to enrollment, and previous treatment with chemotherapy (cyclophosphamide, vincristine, and dacarbazine, CVD), radiopharmaceuticals (MIBG), or tyrosine kinase inhibitors in 8/11 (73%).

However, we found no head-to-head comparison to assess the efficacy of ICIs against other available therapies. Moreover, some additional points should be highlighted.

Firstly, there are a series of ongoing RCTs on both ICI-monotherapy and combination therapy that collectively involve more than 600 patients with solid tumours, including PHEO/PGLs.

Secondly, even if it is presently impossible to draw any conclusion about the antitumoural activity of ipilimumab + nivolumab in PHEO/PGL based on the preliminary results so far available, very recent data suggest the potential benefits of such a combination of drugs in other NENs [48,49]. In addition to current trials on combination therapy, potential future studies employing ICIs in sequence, or as sandwich therapy, would be crucial to further define the role if ICIs in patients with aggressive PHEO/PGL.

Thirdly, the (possible) results on NENs of RCTs employing ICIs not yet approved by FDA for human use (i.e., spartalizumab, a PD-1 inhibitor (RCT *NCT03891953* and *NCT04261439*), and tremelimumab, a PD-1 inhibitor (RCT *NCT02643303*), might hopefully open new horizons in this field.

Fourthly, the identification of potential predictors of response to ICI therapy in patients with diverse solid tumours, including PHEOs/PGLs, is underway. In addition to the above mentioned, radiomic-based predictive model [42], a recent study including 177 PHEOs/PGLs [50] identified, through the analysis of gene expression data of the primary tumours, an algorithm able to predict the positive response to ICIs. Worthy of note, PD-L1 expression by immunohistochemistry has been advocated as a putative predictive correlate of response to anti-PD-1 therapies, and incorporated as a biomarker in clinical studies [51]. Even though evidence that supports this specific hypothesis in PHEO/PGL patients still lacks, it is self-evident that the identification of putative predictors of response will bring into focus the use of ICIs in this setting. 

As a final point, we should consider that PHEO/PGL patients harboring specific mutations might benefit the most from ICIs therapy. In fact, despite most of PHEOs/PGLs are classified as sporadic tumours, germline mutations were identified in about 35% of them, involving over 20 genes [52]. These mutations are classified in three clinically-relevant subgroups: pseudohypoxia, kinase signaling, and Wnt signaling [53]. The pseudohypoxia subgroup includes mutations in a highly significant number of genes, including succinate subunits A, B, C, D, fumarate hydratase, and von Hippel-Lindau tumour suppressor gene, encoding for proteins involved in the cell response induced by conditions of hypoxia. Of note, mutations in this subgroup of genes, although acting at different metabolic levels and on different substrates, ultimately cause the stabilization of the hypoxia-inducible factor (HIF). It was recently reported that increased expression of the immune checkpoint ligand PD-L1 occurs via a HIF-dependent mechanism potentiating the capacity of tumour cells and their surrounding stroma to repress T cells activity [54]. Thus, the engagement of PD-1 signaling represents a novel pathway of T-cell suppression promoted by pseudohypoxia, and provides the rational basis for a more effective use of ICIs in the specific subgroup of PHEO/PGL patients bearing the above mentioned pseudohypoxia-related gene mutations. 

## 5. Conclusions

Published data make a great expectation on the therapeutic use of ICIs in aggressive (progressive/metastatic) PHEO/PGL. New trials are in the starting blocks, and this fervid activity underlines the interest of the scientific community to define the role of a therapy that has already shown outstanding results for other types of cancer. 

## Figures and Tables

**Table 1:** Pharmacokinetic properties of Immune Checkpoint Inhibitors.

Molecule	Commercial Name (FDA Approval)	Target	Antibody Description	Relevant Pharmacokinetic Parameters	Posology for Approved Indications	Notes
Avelumab	Bavencio (March 2017)	Programmed death receptor ligand-1 (PD-L1)	Fully human monoclonal antibody (IgG1-lambda), developed by Merck	Steady state reached after 4–6 weeks (2–3 cycles); volume of distribution is 4.72 L. Terminal half-life is approximately 6.1 days in patients receiving 10 mg/Kg. Total systemic clearence 0.59 L/day	In monotherapy, it is administered e.v.at the dose of 800 mg, as slow infusion (60 min) every 2 weeks	Premedication with antihistamine drugs and acetaminophen is required for the first 4 weeks of treatment.
Atezolizumab	Tecentriq (October 2016)	Programmed death receptor ligand-1 (PD-L1)	Humanized monoclonal antibody (IgG1-kappa), developed by Hoffmann-La Roche AG	Steady state reached after 6–9 weeks. Volume of distribution is 6.91 L. The half life of atezolizumab is 27 days. Total systemic clearence 0.2 L/day	It is admininistered e.v. at the dose of 840 mg/every two weeks	Expression of PD-L1 (>0.1%) has to be demontrated before starting the therapy
Ipilimumab	Yervoy (March 2011)	Cytotoxic T lymphocyte antigen-4 (CTLA-4)	Fully human monoclonal antibody (IgG1-kappa), developed by Bristol-Myers Squibb and Medarex	Steady state reached after 9 weeks. Terminal half life of 15.4 days. Total systemic clearence 0.4 L/day	In monotherapy, it is administered e.v.at the dose of 3 mg/kg, as slow infusion (90 min) every 3 weeks. 1 or 3 mg/Kg can be administered in association with nivolumab (1–3 mg/Kg)	Liver and thyroid function test have to be performed before every administration
Nivolumab	Opdivo (December 2014)	Programmed death receptor-1 (PD-1)	Fully human monoclonal antibody (IgG4-kappa), developed by Bristol Myers Squibb.	Steady state reached after 25 days. Ternimal half life 25 days. Total systemic clearence 0.19 L/day	In monotherapy, it is administered e.v.at the dose of 240 (480) mg, as slow infusion (30–60 min) every 2 weeks	–
Pembrolizumab	Keytruda (September 2014)	Programmed death receptor-1 (PD-1)	Humanized monoclonal antibody (IgG4-kappa), developed by Merck & Co.	Terminal half-life of pembrolizumab is 22 days. Volume of distribution is approximately 6 L. Clearance is 0.22 L/day, and proportionally increases with the body weight.	In monotherapy, it is administered e.v.at the dose of 200 or 400 mg, as slow infusion (30 min) every 3 weeks	Before treatment, the assessment of tumour expression of PD-L1 is recommended
Cemiplimab	Libtayo (September 2018)	Programmed death receptor-1 (PD-1)	Fully human monoclonal antibody (IgG4-kappa), developed by Regeneron Pharmaceuticals	Volume of distribution is 5.2 L. Steady state clearance is 0.2 L/day.The elimination half-life at steady state is 19 days	350 me every 3 weeks, as slow e.v. infusion (30 min)	–
Durvalumab	Imfinzi (May 2015)	Programmed death receptor ligand-1 (PD-L1)	Fully human monoclonal antibody (IgG1-kappa), developed by Astra.Zeneca	Steady state is reached after 16 weeks. Terminal half-life of durvalumab is 18 days. Volume of distribution is 5.64 L. Steady state clearance is 0.2 L/day. Pharmacokinetics is not changed when used in combination with chemotherapy.	it is administered e.v.at the dose of 10 mg/kg every 2 weeks	PD-L1 should be expressed in ≥1% of tumour cell

**Table 2:** Ongoing Registered Clinical Trials.

ClinicalTrials. Gov Identifier	Molecule	Trial Name	Study Phase	Medical Condition under Investigation	Assigned Intervention	Primary Outcome	Estimated Enrollment, *n*	Estimated Study Completion Date	Trial Status
NCT02923466	Avelumab	Phase 1 Trial of Vesicular Stomatitis Virus Genetically Engineered to Express NIS and Human Interferon Beta (VSV-IFNβ-NIS) Monotherapy and in Combination With Avelumab, in Patients With Refractory Solid Tumors	Phase I	Metastatic Colorectal Cancer, Pheochromocytoma, Neuroendocrine Tumours	[arm III]: VSV-IFNβ-NIS will be administered as a single dose on day 1 Avelumab will be administered intravenously every 2 weeks starting on day 1	Maximum tolerated dose of VSV-IFNβ-NIS in monotherapy and combination therapy with avelumab [Time Frame: 21 days after VSV-IFNβ-NIS monotherapy or combination therapy for each dose cohort]	114	July 2021	Recruiting
NCT04400474	Atezolizumab	Exploratory Basket Trial of Cabozantinib Plus Atezolizumab in Advanced and Progressive Neoplasms of the Endocrine System. CABATEN Study	Phase II	Neuroendocrine Tumours, Anaplastic Thyroid Cancer, Adenocarcinoma, Pheochromocytoma, Paraganglioma	Combination of cabozantinib 40 mg + atezolizumab 1200 mg until disease progression, unacceptable toxicity or patient consent withdrawal	Objective response rate [Time Frame: Through study completion, average 1 year]	144	March 2024	Recruiting
NCT02834013	Ipilimumab + Nivolumab	DART: Dual Anti-CTLA-4 and Anti-PD-1 Blockade in Rare Tumors	Phase II	95 listed solid rare tumours, including Pheochromocytoma and Paraganglioma	[arm I]: Nivolumab IV over 30 min on days 1, 15, and 29 + ipilimumab IV over 60 min on day 1. Treatment repeats every 42 days for up to 17 cycles in the absence of disease progression or unacceptable toxicity. [arm II]: Nivolumab IV over 30 min on days 1, 15, and 29. Treatment repeats every 42 days for up to 17 cycles in the absence of disease progression or unacceptable toxicity.	Overall response rate defined as confirmed and unconfirmed complete and partial response [Time Frame: Up to 10 years]	818	No date given (Estimated Primary Completion Date: August 2021)	Recruiting
NCT03333616	Ipilimumab + Nivolumab	A Phase II Study of Nivolumab Combined With Ipilimumab for Patients With Advanced Rare Genitourinary Tumors	Phase II	Genitourinary Cancer, Adrenocortical Carcinoma, Non-urothelial Bladder Cancer Non-urothelial Upper Tract Penile Cancer, Non-adenocarcinoma Prostate Cancer, Refractory Germ-cell Cancer, High Grade Neuroendocrine Carcinoma, Small Cell Carcinoma, Paraganglioma	Nivolumab and Ipilimumab administered every 3 weeks for a total of 4 maximum doses. After combination therapy, nivolumab will be administered as monotherapy every 4 weeks.	Objective Response Rate [Time Frame: Imaging will occur every 6–12 weeks study entry up until disease progression (up to 24 months)]	57	May 2025	Recruiting
NCT04187404	Nivolumab	A Phase 1/2 Trial of a Novel Therapeutic Vaccine (EO2401) in Combination With Immune Check Point Blockade, for Treatment of Patients With Locally Advanced or Metastatic Adrenocortical Carcinoma, or Malignant Pheochromocytoma/Paraganglioma	Phase I/II	Adrenocortical Carcinoma, Pheochromocytoma, Paraganglioma	Multiple dose of EO2041 in combination with nivolumab	Adverse events assessment [Time Frame: Up to 24 months]	72	March 2024	Recruiting
NCT02721732	Pembrolizumab	Phase II Study for the Evaluation of Efficacy of Pembrolizumab (MK-3475) in Patients With Rare Tumors	Phase II	17 listed solid rare tumours, including advanced/metastatic/unresectable Pheochromocytoma and Paraganglioma	Pembrolizumab IV over 30 min on day 1. Treatment repeats every 21 days for up to 24 months in the absence of disease progression or toxicity. Patients with clinical response or disease stabilization may continue treatment for up to an additional 12 months.	Non-progression rate (defined as the proportion of subjects in the analysis population who have no progression of disease) [Time Frame: At 27 weeks] Incidence of adverse events [Time Frame: Up to 27 weeks]	225	Decembrer 2020	Recruiting
NCT02332668	Pembrolizumab	A Phase I/II Study of Pembrolizumab (MK-3475) in Children With Advanced Melanoma or a PD-L1 Positive Advanced, Relapsed or Refractory Solid Tumor or Lymphoma (KEYNOTE-051)	Phase I/II	Melanoma, Lymphoma, Classical Hodgkin Lymphoma, Microsatellite-instability-high Solid Tumour, Solid tumours including Pheochromocytoma and Paraganglioma	Pembrolizumab, starting dose 2 mg/kg (maximum dose 200 mg), IV once every 3 weeks.	Objective response rate [Time Frame: Up to 2 years] Number of participants with Dose-Limiting Toxicities [Time Frame: Cycle 1 (Up to 21 days)] Number of participants experiencing Adverse Events [Time Frame: Up to 27 months] Number of participants discontinuing study drug due to Adverse Events [Time Frame: Up to 2 years] Objective response rate by International Working Group (IWG) Response Criteria (Cheson, 2007) per Blinded Independent Central Radiology Assessment [Time Frame: Up to 2 years]	320	September 2022	Recruiting

## Data Availability

No new data were created or analyzed in this study. Data sharing is not applicable to this article.

## Abbreviations

AE: Adverse Event; AR: Adverse Reaction; APC: Antigen-Presenting Cell; BR: Best Response; CBR: Clinical Benefit rate; CTLA-4: Cytotoxic T Lymphocyte Antigen-4; DoR: Duration of Response; HIF: Hypoxia-Inducible Factor; ICI: Immune Checkpoint Inhibitor; MCH: Major Histocompatibility Complex; MTD: Maximum Tolerated Dose; MIBG: Metaiodobenzylguanidine; NPR: Non-Progression Rate; NEN: Neuroendocrine Neoplasm; NSCLS: Non-Small Cell Lung Cancer; ORR: Objective Response Rate; OS: Overall Survival; PD: Progressive Disease; PD-1: Programmed Cell Death-1; PD-L1: Programmed Death-Ligand-1; PD-L2: Programmed Death-Ligand-2; PFS: Progression-Free Survival; PGL: Paraganglioma; PHEO: Pheochromocytoma; PR: Partial Response; RCT: Registered Clinical Trial; SD: Stable Disease; TAA: Tumour-Associated Antigen; TCR: T-Cell Receptor; TIL: Tumour Infiltrating Lymphocyte; VV1: VSV-IFNβ-NIS.
